# Performance of commonly used respiratory questionnaire items in a cohort of infants born preterm

**DOI:** 10.4236/ojped.2013.33045

**Published:** 2013-09-01

**Authors:** Elizabeth Boggs, Nori Minich, Anna Maria Hibbs

**Affiliations:** 1 School of Medicine, Case Western Reserve University, Cleveland, USA; 2 Department of Pediatrics, Case Western Reserve University, Cleveland, USA; 3 Division of Neonatology, Rainbow Babies and Children’s Hospital, Cleveland, USA

**Keywords:** Respiratory Questionnaire, Preterm, Wheeze

## Abstract

**Background:**

Items from respiratory questionnaires validated in older children are often used in research studies of preterm infants, although they have not been validated in this population. We aimed to assess both test-retest reliability and convergent validity of a group of commonly used respiratory questionnaire items in a cohort of preterm infants.

**Methods:**

The health status of 300 preterm infants was assessed by telephone questionnaire as part of a prospective cohort study. The questionnaire items analyzed in this study included six commonly used respiratory questions. The questionnaire responses used in this analysis were from the telephone follow-up in this cohort at six months of age adjusted for prematurity. A repeat interview one to two weeks after this interview was performed in a subset of subjects to assess test-retest reliability. The convergent validity of the respiratory items was also assessed by calculating the associations among the responses to the respiratory questions.

**Results:**

A total of 43 infants were singletons that met the criteria for test-retest reliability analysis. All of the respiratory questions demonstrated fair to strong test-retest reliability. Among 206 respondents, respiratory questionnaire items also demonstrated strong convergent validity, in that caretakers reporting wheezing or whistling in the chest were significantly more likely to also report other respiratory events.

**Conclusions:**

This selection of standard respiratory questionnaire items performed well for research purposes in this population.

## 1. INTRODUCTION

Persistent wheezing in the first year of life is a common and long-lasting complication of prematurity [1,2,3–7]. Preterm infants born at less than 37 weeks gestational age (GA) are more likely than term-born infants to experience wheezing in infancy and also to develop asthma [2,4,8–12]. While several questionnaires assessing respiratory health have been well-validated in older children, their performance in infants, and in particular preterm infants, have not been well assessed [13–19].

Non-invasive questionnaire-based assessments of the respiratory outcomes of neonatal intensive care unit (NICU) graduates often borrow from validated questionnaires designed for older children, often those with asthma. For instance, in the follow-up of trials testing interventions to decrease bronchopulmonary dysplasia, parent-perceived wheezing is often assessed by asking about “wheezing and whistling in the chest”, a phrasing borrowed from questionnaires such as the International Study of Asthma and Allergies (ISAAC) questionnaire [17,18] and American Thoracic Society child questionnaire ATS-DLD-78-C [19]. In addition, parental reports of medical interventions, such as hospitalization, physician diagnoses, or prescription of medications, are also frequently used as markers of the long-term pulmonary status of infants born preterm [20–26]. Such questions have been used in the follow-up assessments of major multi-center trials studying preterm infants (e.g. NO CLD [NCT00006401] [14,20] and SUPPORT [NCT-00233324]) [21,22,27]. However, the reliability and validity of these questions in the premature population have not been established.

The objective of this study was to assess the performance of a group of commonly used respiratory questionnaire items in a preterm cohort. We aimed to both assess the test-retest reliability and explore the convergent validity of this set of questionnaire items. We hypothesized that the items would demonstrate good test-retest reliability and that the responses to questions about respiratory morbidity, such as parental reports of “wheezing or whistling in the chest” or inhaled medication use, would be highly correlated with each other.

## 2. METHODS

### 2.1. Population

As part of a prospective cohort study of 300 preterm infants, “Gastrointestinal Risk Factors for Wheezing in Premature Infants (GRWPI)”, health status was assessed by telephone questionnaire at 3, 6, 9, and 12 months of age adjusted for prematurity. This study enrolled infants born at 28^0/7^ – 34^6/7^ GA. Infants with bronchopulmonary dysplasia (supplemental oxygen requirement >28 days), major anomalies, severe neurological injury, or a history of necrotizing colitis were excluded from the study. All participants were born at or transferred to Rainbow Babies and Children’s Hospital.

### 2.2. Performance of Questionnaire Items

This analysis of the performance of the respiratory items on the questionnaire focused on the assessment at 6 months adjusted age. At this time point, a repeat interview was planned one to two weeks after the primary interview in the first 60 infants that were followed. The analysis of test-retest reliability was restricted to singletons for whom the same caretaker participated in both interviews.

The questionnaire items analyzed in this study included the following:

“Has your child had wheezing or whistling in the chest?”
“Has a doctor diagnosed your child with wheezing or asthma?”
“Has your child had a respiratory infection, such as a cold, bronchiolitis, or pneumonia?”
“Has your child been diagnosed with RSV, or Respiratory Syncytial Virus?”
“Has your child been seen in the emergency room for a breathing problem?”
“Has your child been admitted to the hospital for a breathing problem?”

Answer choices were “yes,” “no,” and “unsure”. “Unsure” answers were treated as missing.

### 2.3. Convergent Validity

Odds of reporting a concurrent respiratory event given positive responses to another respiratory questionnaire item were calculated to assess internal consistency and convergent validity. An additional non-respiratory questionnaire item “In the past week, has your child refused a feed when hungry?” was included as a control.

### 2.4. Statistical Analysis

Test-retest reliability was assessed using both Cohen’s kappa and percent agreement. Associations between markers of respiratory morbidity (e.g. hospitalization and medication use) were assessed with odds ratios, Chi square’s, and Fischer’s exact tests. Statistical analyses were performed with Stata version 9.2 (StataCorp, College Station, TX).

## 3. RESULTS

Two hundred seventy nine infants were followed at the six month time point (93% follow up). Of these, 206 were singletons meeting criteria for the analysis. The chronological age of participants at this interview ranged from 206 to 278 days (Table 1).

Test and retest respiratory interviews were conducted in 61 infants, as opposed to the goal of 60, due to the presence of twins in the sample. Among the 61 infants, 43 met criteria for the test-retest reliability analysis. Demographic characteristics for the test-retest subset were generally representative of the whole cohort (Table 1). Repeat administrations of the questionnaire were done 6 – 19 days after the first administration.

In this relatively healthy population of preterm infants, several of the questions had a low rate of positive responses. Doctor diagnoses of wheezing or asthma, RSV infections, ER visits for breathing problems, and hospital admissions for breathing problems were the least frequently reported markers of respiratory morbidity. Wheezing and whistling in the chest and respiratory infections had the highest reported rates (Table 2).

All of the respiratory questions demonstrated fair to strong test-retest reliability (Table 2). The strongest agreement existed for doctor diagnosis of wheezing or asthma, hospital admissions for breathing problems, and RSV infections. ER visits for a breathing problem and wheezing and whistling in the chest also demonstrated substantial agreement.

Convergent validity was assessed by calculating the odds of a caretaker answering an item affirmatively if they had also answered another item affirmatively (Table 3). Notably, caretakers reporting parental recognition of wheezing or whistling in the chest were significantly more likely to also report a doctor diagnosis of wheezing, respiratory infection, RSV infection, ER visit for a breathing problem, hospitalization, and use of inhaled or use of oral steroids to address a breathing problem. Refusal to feed was significantly correlated with wheezing and whistling in the chest and doctor diagnosis of RSV infection, though overall it was not strongly correlated with this set of respiratory questions.

## 4. CONCLUSIONS

We assessed the performance of commonly used respiratory questionnaire items in a cohort of relatively healthy preterm infants. All of the respiratory questions demonstrated fair to strong test-retest reliability. Furthermore, these items demonstrated convergent validity; the markers of respiratory severity assessed were strongly associated with each other. Taken together, these results suggest that these standard respiratory questions perform well for research purposes in this population.

In the analysis of test-retest reliability, respiratory events with lower rates of positive responses tended to demonstrate better agreement. This is likely due to the fact that the less frequent events, such as hospitalization, may also be more salient for the parent. However, rare outcomes are non-ideal primary endpoints for research studies due to their impact on sample-size. Furthermore, hospitalization may be driven by a number of non- medical factors [28–35]. The more frequently reported parental recognition of symptomatic wheezing or whistling in the chest may be an outcome prone to more “noise” from misclassification; however, in real-world clinical interactions, physicians often rely on parental reports of symptom burden to drive care. Thus, studies wishing to assess respiratory morbidity by parental questionnaire benefit by capturing several complementary outcomes, which, as we have shown, do demonstrate strong convergent validity.

Interestingly, doctor diagnosis of wheezing or asthma performed very well in terms of test-retest reliability, yet it was the only item that was not significantly associated with other respiratory events in the exploration of convergent validity. One possible reason for this outcome is that the question “Has a doctor diagnosed your child with wheezing or asthma?” is a compound question, in that wheezing and asthma are two different diagnoses. A compound question was asked instead of a traditional wording validated in older children about doctor-diagnosed asthma due to reluctance of many practitioners to differentiate between wheezing and asthma in infants less than one year old. However, this may have weakened the performance of the question. Another possible reason for this outcome is that it reflects a true lack of association between doctor diagnosis and the other items assessed, perhaps due to a different threshold for using or recalling symptomatic treatment versus the application of a diagnostic label. Perhaps unsurprisingly, refused feed was correlated with wheezing and whistling in the chest and diagnosis of RSV infection, both indicators of symptomatic illness. Overall, this question did not correlate significantly with most of the respiratory questions, an indicator that correlation among respiratory items was not primarily driven by a general tendency of parents to affirm symptoms.

This study has several limitations. While these respiratory questions perform well in terms of test-retest reliability and convergent validity, it is noteworthy that the responses are not matched against medical records, thus we cannot assess the accuracy of these results. Furthermore, this analysis is a limited assessment of the performance of a series of commonly used respiratory questionnaire items. While it represents a first step in the assessment of the performance of these items in the preterm population, it is not a full validation of a freestanding questionnaire.

Nevertheless, this study offers reassurance that questionnaire items commonly used to asses respiratory outcomes in preterm infants perform well in terms of test- retest reliability and convergent validity. Reliable and valid non-invasive measures are ideal assessment tools in a medically fragile population in whom more invasive testing of pulmonary status may be both medically risky and expensive. Furthermore, respiratory symptoms and resource utilization may reflect the experience of patients and families better than physiologic measures. Though this study is an initial step in fully validating a questionnaire in this population, so far the performance of these commonly used items suggests that their use in studies of preterm infants is sound. Future work is needed to develop a fully validated questionnaire to standardize the assessment of respiratory status in preterm infants.

## Figures and Tables

**Table 1:** Baseline characteristics of the infants and parents.

	Median (Range)	

Infant Characteristics	Entire 6 Month Cohort (n = 206)	Test-Retest Subset (n = 43)
Birth weight, g	1725 (870 – 3150)	1570 (1073 – 2630)
Gestational age, weeks	32 (28 – 34)	32 (28 – 34)
Days on oxygen	0 (0 – 23)	0 (0 – 12)
Days on ventilation	0 (0 – 5)	0 (0 – 5)
Age at 6-month follow up survey, days	240 (206 – 278)	244 (217 – 278)
**Parental characteristics**	N (%) (n = 206)	N (%) (n = 43)
Maternal Race		
White	87 (42.2%)	23 (53.5%)
Black/African American	112 (54.4%)	17 (39.5%)
Other	7 (3.4%)	3 (7.0%)
**Paternal Race**		
White	85 (41.3%)	21 (48.8%)
Black/African American	115 (55.8%)	19 (44.2%)
Other	6 (2.9%)	3 (7.0%)

**Table 2:** Kappa scores and percent agreement for the test-retest reliability of the respiratory questionnaire.

Respiratory Questions	Percent that Responded “Yes” in the First Interview	Kappa	Percent Agreement
Has your child had a respiratory infection?	53.7%	0.40	69.8%
Has your child had wheezing or whistling in the chest since our last interview?	38.5%	0.73	88.4%
Has your child been seen in the ER for a breathing problem?	14.2%	0.88	97.8%
Has a doctor diagnosed your child with wheezing or asthma?	7.8%	1.0	100%
Has your child been admitted to the hospital for a breathing problem?	6.3%	1.0	100%
Has your child been diagnosed with RSV?	4.9%	1.0	100%

**Table 3:** Internal consistency and convergent validity. The odds ratios for reporting a respiratory event given a report of another event are listed.

	Odds of Reporting Respiratory Event OR [95% CI] p-value								

	Wheezing or Whistling in the Chest	Doctor Diagnosis of Wheezing	Respiratory Infection	RSV	ER visit	Hospital Admission	Inhaled Medication	Oral Steroids	Refused Feed
Wheezing or Whistling in the Chest		* p < 0.0001	4.34 [2.34, 8.06] p < 0.0001	** p < 0.0001	20.1 [5.83 – 69.3] p < 0.0001	10.0 [2.16, 46.6] p = 0.0004	26.5 [7.73, 90.7] p < 0.0001	26.9 [3.46, 209] p < 0.0001	2.75 [1.27, 5.96] p = 0.0084
Doctor Diagnosis of Wheezing			1.50 [0.523, 4.29] p = 0.4487	3.18 [0.615, 16.4] p = 0.1822	10.8 [3.63, 32.2] p < 0.0001	2.54 [0.507, 12.8] p = 0.2398	142 [17.7, 1140] p < 0.0001	9.04 [2.59, 31.6] p < 0.0001	1.90 [0.573, 6.33] p = 0.2864
Respiratory Infection				** p = 0.0020	3.92 [1.52, 10.1] p = 0.0028	*** p = 0.0005	5.07 [2.00, 12.9] p = 0.0002	13.7 [1.77, 106] p = 0.0014	1.80 [0.819, 3.96] p = 0.1395
RSV					18.1 [4.37, 75.3] p < 0.0001	7.84 [1.76, 35.0] p = 0.0191	5.66 [1.54, 20.8] p = 0.0134	18.3 [4.54, 73.7] p < 0.0002	3.93 [1.04, 14.8] p = 0.0041
ER Visit						6.30 [1.95, 20.4] p = 0.0006	16.4 [6.59, 40.6] p < 0.0001	26.3 [7.58,91.1] p < 0.0001	1.51 [0.559, 4.05] p = 0.4126
Hospitalization							23.3 [5.99, 90.8] p < 0.0001	26.8 [7.31, 98.5] p < 0.0001	1.69 [0.437, 6.50] p = 0.4323
Inhaled Medication								52.3 [11.0, 248] p < 0.0001	1.88 [0.765, 4.65] p = 0.1636
Oral Steroids									1.39 [0.369, 5.22] p = 0.7095
Refused Feed									

* Odds ratio could not be calculated because there were no infants for whom the parents reported a diagnosis of wheezing but no wheezing symptoms;

** Odds ratio could not be calculated because there were no infants for whom the parents reported a diagnosis of RSV but no wheezing symptoms;

*** Odds ratio could not be calculated because there were no infants for whom the parents reported a respiratory hospitalization but no respiratory infection.
